# The Polish version of the Cultural Intelligence Scale: Assessment of its reliability and validity among healthcare professionals and medical faculty students

**DOI:** 10.1371/journal.pone.0225240

**Published:** 2019-11-25

**Authors:** Krystian Barzykowski, Anna Majda, Małgorzata Szkup, Paweł Przyłęcki

**Affiliations:** 1 Institute of Psychology, Jagiellonian University, Kraków, Poland; 2 Faculty of Health Sciences, Institute of Nursing and Midwifery, Jagiellonian University Medical College, Kraków, Poland; 3 Department of Nursing, Pomeranian Medical University in Szczecin, Szczecin, Poland; 4 Faculty of Health Sciences, Department of Sociology, Medical University of Lodz, Łódź, Poland; Warszawski Uniwersytet Medyczny, POLAND

## Abstract

**Background:**

Healthcare professionals and students of medical faculties in Poland increasingly encounter culturally diverse patients. It is necessary to support the development of cultural intelligence in order to improve the medical care provided to patients from different cultural backgrounds. At present there are no standardized tools in Poland that can accurately and reliably assess cultural intelligence, which is defined by Ang et al. as “*an individual’s capability to function and manage effectively in culturally diverse settings*”. As argued in the present paper, this (cap)ability may be important for providing patient-centred care that is culturally adequate and competent.

**Purpose:**

The aim of the research was to show the multistage process of validation of the Polish version of The Cultural Intelligence Scale by Ang et al. and Van Dyne et. al.

**Methods:**

Across two studies we examined the psychometric properties of the Cultural Intelligence Scale, including reliability (i.e. internal consistency, test-retest reliability, factor structure) and validity (i.e. theoretical, criteria, convergent). In the first two-session study, 349 participants (98% were healthcare professionals, e.g. nurse, student nurse, medical student; mainly women, 89%) completed the Polish version of the Cultural Intelligence Scale twice with an interval of at least 22 days. In addition, across two study sessions participants completed questionnaires constructed to measure (a) cultural competence, (b) need for cognitive closure, (c) emphatic sensitiveness, (d) emotional intelligence, (e) self-esteem, (f) social desirability, (g) personality, and (h) positive/negative attitudes towards culturally divergent people. Finally, to additionally examine the theoretical validity, 36 professional cross-cultural competence trainers completed the Cultural Intelligence Scale during a one-session study.

**Results:**

The Cultural Intelligence Scale has been shown to have satisfactory psychometric properties. It has high reliability (Cronbach’s alpha, respectively .94 and .95 in the first and second sessions) and the factor structure seems to approach the postulated one. Theoretical and criterion accuracy are well proven; convergence is less straightforward, but it correlates well with tools that examine variables such as cultural competence, cognitive closure, empathy/emphatic sensitiveness, emotional intelligence, self-esteem, personality, and social desirability. The results suggest that these factors contribute to the development of the cultural intelligence.

**Conclusion:**

The Cultural Intelligence Scale can be successfully used in empirical research of cultural intelligence of medical professionals and students of medical majors and their education in Polish conditions.

## Introduction

The concept of cultural intelligence is a relatively new construct from the early 21st century that is well described in English-language literature. The term ‘cultural intelligence’ is known in business and management of multicultural international teams and social psychology [1, 2, 3, 4, 5, 6, 7, 8, 9], but it is slightly less known in health care [10, 11]. In Polish literature, cultural intelligence is a concept that is increasingly encountered, for example in management [12, 13, 14, 15, 16], or it is almost not used at all, e.g. in medicine and nursing [17]. However, cultural intelligence as a core skill of cultural competence is very important in contemporary cross-cultural healthcare.

Until recently, medical and nursing students in Poland were not trained in cultural competencies as these were not, unfortunately, considered essential for healthcare professionals in Poland due to the ethnically homogenous nature of Polish society. However, Poland’s accession to the EU in 2004 changed this situation dramatically and in recent years this country has observed a rapid growth of foreigners working or studying there [18]. In 2011, foreigners permanently living in Poland made up only 0.2 percent of the population [19], but by 2018 this number had rapidly increased to 1.8 percent [20]. However, these statistics do not cover unregistered foreigners such as travellers who are in Poland for longer or shorter periods of time. These changes in the structure of Polish society led to the necessity of implementing cross-cultural competences training in medical curricula, and in fact this is now a legal requirement in Poland [21].

It is worth highlighting that training healthcare professionals in cultural competence is deemed necessary by the US Department of Health and Human Services [22]. This US Department proposed 14 standards of culturally competent medical care. Culturally sensitive care means striving to overcome linguistic, cultural and communication barriers in the provider–patient relationship. It is known–mostly in the holistic model of treatment–that “culture, spirituality, and religion, as methods of an approach to life influence the lifeworld experience in a special way with regard to health and illness, dignity, autonomy, moral feeling, and the handling of life and death” ([23], p. 229). Lack of cultural sensitivity results in miscommunication between healthcare providers and patients. The ethnocentric attitude (i.e. a tendency to see and perceive other groups or cultures from the perspective of one’s own culture) of health care professionals may impede the understanding of a patient. For example, people from different cultures can report pain differently [24]. Importantly, the whole process of clinical assessment (observation, history-taking, physical examination, laboratory testing) may be affected by culture [25].

According to the patient-centred care model, an individual patient’s needs, values and preferences should be respected [26]. Thus, healthcare professionals should strive to go beyond their own cultural frames and perspective in order to build an atmosphere of trust in the provider–patient relationship. Therefore, health care professionals characterized by a high level of cultural intelligence should be aware of the importance of a patient’s culture in clinical assessments. They should know both how to communicate with patients of different social and/or cultural backgrounds and to encourage them to express their spiritual beliefs or cultural practises [27]. Culturally sensitive healthcare professionals have some knowledge about minorities in that they understand their values and use culturally appropriate language [24]. For all these reasons, cultural intelligence should be considered an important and valuable ability in the healthcare context.

### Cultural intelligence

Initially, Earley and Ang [5] proposed a concept of cultural intelligence that consisted of the three following components: cognitive, behavioural and motivational. The concept was then developed by, among others, Earley and Mosakowski [6], Ang et al. [1], Ang et al. [2], Van Dyne et al. [8], and Van Dyne et al. [9]. To put it simply, cultural intelligence, or CQ in short, is an individual’s ability to recognize the rules of an unknown social environment, and then to absorb them and apply them effectively in a new culturally diverse environment. Cultural intelligence is consistent with the theory of general intelligence formulated by Stern, the author of the IQ intelligence quotient, who understood it as an individual’s ability to adapt to the surrounding environment. It also has many features in common with emotional intelligence, but it is a somewhat broader concept that includes, for example, the ability to distinguish culturally determined behaviours from behaviours that are manifestations of the individual personality traits that are typical of all people regardless of cultural background.

Based on the works of Ang et al. [1] and Van Dyne et al. [8], CQ is a structure that consists of 4 components: motivational, cognitive, metacognitive, and behavioural. The motivation component of CQ emphasizes authentic interest in other cultures and interactions with their representatives. The cognitive component of CQ includes knowledge of other cultures’ norms, values, principles, beliefs, customs, rituals, symbols, ceremonies, habits, gestures and cultural artefacts; it also covers economic, legal, health care, and education systems, as well as behaviours that do not violate social norms. The cognitive element is the ability to understand and interpret information in a given cultural context. The metacognitive component of CQ is responsible for the awareness of differences between cultures and understanding the beliefs of other people through the prism of their culture; this helps to perceive and understand behaviours adopted in a different culture. The behavioural component of CQ relates to the ability to behave according to the norms of other cultures; it enables unknown rules of conduct to be mastered and effective operation in different cultural conditions. A culturally intelligent person understands which behaviours are applied in different cultures and effectively cooperates with representatives of these cultures.

According to Starosta [16], cultural intelligence is a specific form of intelligence that represents an individual’s ability to understand and function properly in situations that are characterised by cultural diversity. According to Piwowarczyk [12], cultural intelligence enables adaptation and smooth functioning in a new cultural environment; it is a human potential–a seemingly inborn personality trait that can be identified, assessed, and developed in the university education process. According to Simpson [15], the development and improvement of cultural intelligence is quite a long process that is difficult but can bring satisfaction as it facilitates the overcoming of stereotypes that hinder relations between representatives of different cultures.

Cultural Intelligence is a skill worth transferring to the management of teams caring for patients from different cultural backgrounds or multicultural teams dealing with patients. In addition, cultural intelligence seems to be a crucial skill that also needs to be developed by students of medical majors in the process of academic education, including nursing in Poland [17]. Understanding the essence, structure, components and significance of cultural intelligence and developing methods to measure it is an important and necessary element of implementing the idea of multiculturalism in medical education in Poland. This can be helped by English-language tools for measuring cultural intelligence, such as *the Cultural Intelligence Scale (CQS)* by Ang et al. [1, 2] and Van Dyne et. al. [8, 9]. The authors of the article recommend the usefulness of the issues described in nursing and medicine in relation to the increasingly frequent contact of Polish nurses, doctors and students of medical faculties with culturally diverse patients [28]. They postulate measuring and developing the cultural intelligence of nurses and other professionals in the medical sector as an important ability/skill that comprises professional cultural competences. Similarly, Rahimaghaee and Mozdbar [11] emphasize the relationship between cultural intelligence and the professional competence of nurses.

## The present study

Given the fact that to the best of the authors’ knowledge there are no Polish tools (e.g. questionnaires, scales) allowing measurement of cultural intelligence, an overriding goal of the present study was to describe for the first time the translation, adaptation, and psychometric assessment of the Polish version of the Cultural Intelligence Scale (CQS), Ang et al. [1, 2] and Van Dyne et. al. [8, 9]. Ang et al. [1] defined CQ as an individual’s ability to effectively cope with situations characterized by cultural diversity. CQS was developed on the basis of the theoretical model of cultural intelligence of Earley and Ang [5], which consists of four components: metacognitive, cognitive, motivational and behavioural CQ. Metacognitive CQ refers to processes by which individuals acquire and understand cultural knowledge; cognitive CQ is general knowledge about culture; motivational CQ is the amount and direction of energy used in learning and functioning in intercultural situations; Behavioural CQ is the ability to demonstrate appropriate actions when interacting with people of different cultures. The scale consists of 20 items and is characterized by good reliability indicators (αs = .70–.86) and accuracy.

To adapt the CQS to Polish culture, we first carefully translated it and then assessed its psychometric properties. First, we validated the CQS’s reliability in terms of internal consistency (Cronbach’s α), test-retest reliability and its factor structure. Second, we evaluated the CQS’s theoretical, criterion and convergent validity. More precisely, to address theoretical validity we developed The Positive/Negative Attitude Towards Culturally Divergent People Questionnaire. It was expected that people who have not interacted in the past with culturally divergent people would score lower on the CQS compared to participants who have experience in this area and have a positive attitude towards this group. In addition, CQS was completed by professional cross-cultural competence trainers. We expected them to score higher on the CQS compared to the non-professional group of participants. In order to further examine the criterion validity, we compared participants’ scores on the Cross-Cultural Competencies Inventory (CCCI) [29, 30]. We expected these two tools to be highly positively correlated.

Finally, we further investigated the relationship between cultural intelligence and other variables that are expected to be correlated with cultural intelligence. For instance, a number of studies suggest a relationship between CQS and personality traits [1, 2, 31], emotional intelligence [2, 32, 33], leadership effectiveness [34], and cooperative negotiations [35]. Therefore, as a part of examining the convergent validity of the CQS we further investigated the relationship between the cultural intelligence score and factors such as (a) need for cognitive closure, (b) emphatic sensitiveness, (c) emotional intelligence, (d) self-esteem, and (e) personality. It can be argued that these factors may play an important role in the development of cultural intelligence.

For instance, the need for cognitive closure relates to an individual’s preference for closure when making decisions and judgements. Since people with a high need for cognitive closure are rather cognitively closed-minded (i.e. resistant to disconfirmation and ambiguity) and, importantly, in social situations experience discomfort when facing ambiguity because they are rather resistant to change, these traits should be negatively correlated with cultural intelligence, which relates to one’s capability to deal with culturally divergent and therefore ambiguous situations. At the same time, low need for cognitive closure may make individuals behave in a more flexible and therefore less stereotyped and less prejudiced fashion. For instance, they are less prone to misinterpreting everyday situations [36]. Since they may be more tolerant to experiencing uncertainty in social situations and less inclined to quickly form judgments, they may also be more open towards acquiring new information, especially about culturally divergent people.

As for emotional intelligence, it refers to “the ability to perceive emotions, to access and generate emotions so as to assist thoughts, to understand emotions and emotional knowledge and to reflectively regulate emotions so as to promote emotional and intellectual growth” [37]. Since emotional intelligence, for instance, makes it possible to recognize and feel the emotions expressed by others and, importantly, to successfully communicate with people who have different styles of emotional functioning and expression, it is expected to positively correlate with cultural intelligence. Put differently, interacting with a culturally divergent individual may require adequate adaptation to a different way of both communicating and expressing emotions [38]. Similarly, empathy should be also correlated with cultural intelligence. More precisely, empathy refers [39], for example, to the ability to adopt the perspectives (or points of view) of other people, the ability to respond emotionally to observed emotionality (e.g. negative experiences) in others, or to one’s tendency to experience anxiety or discomfort when observing others’ negative experiences. Therefore, a high level of empathy should facilitate going beyond one’s own ‘self’ when interacting with other people by adopting a different point of view and empathizing with others. Importantly, these characteristics may be considered crucial in effectively and adequately interacting with culturally diverse individuals and in developing professional intercultural competence [40, 41]. Thus, we expected these characteristics to be positively correlated with cultural intelligence, while we expected personal distress to correlate negatively.

Next, we expected self-esteem, i.e. one’s attitude towards oneself, to be rather positively correlated with cultural intelligence. This is especially true because people with high self-esteem are more prone to experiencing more positive emotions and are more active and persistent when facing difficulties, challenges and risks [42]. Given the fact that encountering a culturally diverse person may be experienced as a difficult and challenging situation [43], high self-esteem may be especially helpful in behaving in culturally diverse situations.

Finally, we also wanted to examine the relationship between personality traits and cultural intelligence. More precisely, we expected a positive correlation between cultural intelligence and personality traits such as extraversion, agreeableness, emotional stability and intellect [44, 45]. For instance, intellect (in relation to an individual’s openness to new and novel experiences), extraversion (an individual’s level of sociability and social confidence) and agreeableness (having a positive attitude towards other people) may be important for engaging in cross-cultural communication and interpersonal relationships.

In summary, in the present study we wanted to thoroughly and carefully examine the reliability and validity of the Cultural Intelligence Scale. In addition, we wanted to verify the expected relationship between cultural intelligence and other factors such as empathy, sensitiveness, need for cognitive closure, emotional intelligence, self-esteem and personality. As argued above, these factors should contribute to the development of cultural intelligence.

## Method

### Design

A mixed-subject design was employed in the present study. We analysed the differences between a group of non-cross-cultural trainers and a group of cross-cultural trainers in terms of their total score in the CQS (between-subject factor). At the same time, the other variables were treated as within-subject factors (e.g. the total score of CQS in the first and second session).

### Participants

A total of 349 individuals (called also non-cross-cultural trainers or non-professionals) aged 18–53 participated in the study, mainly women (312 female, 37 male; M = 21.49, SD = 4.72). All indicated Polish nationality. The majority of participants (343, around 98%) were healthcare professionals (around 27%; e.g. nurse, medical), medical students (around 23%) and student nurses (around 47%). Participants (medical student, student nurse, medical student, nurse or doctor) were recruited from two large medical universities in Poland; namely, Jagiellonian University Medical College and The Medical University of Lodz. In this way (i.e. by including two different universities) we wanted to increase the representativeness of the sample of the population of healthcare professionals, medical students and student nurses in Poland. Importantly, 75% of participants were recruited from Jagiellonian University and 25% were recruited from the Medical University of Lodz. We included in the final sample only individuals who (1) indicated Polish nationality, (2) participated in both sessions, and (3) filled in all materials provided during each session. Therefore, based on these inclusion criteria, 32 participants were excluded from the final sample.

Participants completed two sessions, each one on separate days (average distance = 28.06 ±4.40 days, range = 22 to 47 days). To keep the sessions as comparable as possible in terms of the time of the day and activities, the second session was scheduled at least 22 days later, at the same time and day as the previous one whenever possible.

Moreover, a total of 36 professional cross-cultural competence trainers aged 28–65 participated in the one-session study (26 females, 10 males) (M = 45.66, SD = 8.61, two participants did not indicate their age). All these participants finished a 250-hour Training the Trainers in Multicultural Education and Competences course organized by the Polish Helsinki Human Rights Foundation (HHRF) and are officially recommended by the HHRF as professional cross-cultural competence trainers.

### Instrument

#### The Cultural Intelligence Scale

The Cultural Intelligence Scale (CQS) [2] is a self-report measure concerning an individual’s (cap)ability to effectively cope with situations characterized by cultural diversity. Therefore, it may be argued that cultural intelligence as measured by the CQS should be treated as a (cap)ability rather than as personality trait(s) and/or competence(s). Participants are asked to read each statement and select the answer that best describes their capabilities using a 7-point scale (1 = *strongly disagree*; 7 = *strongly agree*; the higher the degree of agreement, the higher the degree of cultural intelligence in general, and in particular within a given domain). The CQS consists of 20 statements (total score ranging from 20 to 140) covering four components of Cultural Intelligence:

1. Metacognitive CQ (4 items, score ranging from 4 to 28; e.g. *I am conscious of the cultural knowledge I apply to cross-cultural interactions*).
2. Cognitive CQ (6 items, score ranging from 6 to 42; e.g. *I know the legal and economic systems of other cultures*).
3. Motivational CQ (5 items, score ranging from 5 to 35; e.g. *I enjoy interacting with people from different cultures*).
4. Behavioural CQ (5 items, score ranging from 5 to 35, e.g. *I change my verbal behaviour (e*.*g*., *accent*, *tone) when a cross-cultural interaction requires it*).

The CQS was translated into Polish by two independent translators with high proficiency in English. The translations were then evaluated and adjusted to the final version of the inventory by three of the authors of this paper (K.B., A.M., and P.P.). The final translation was subsequently back-translated into English by an independent translator with high proficiency in English. The back-translated version was then evaluated by the three authors of the present study (K.B., P.P., and M.S.). Any differences between the original and back-translated version of the CQS were resolved by discussion and the final version of the CQS was amended accordingly and revised by A.M. Please note that on the 5th of December 2017 the second author of the present paper (AM) obtained the authors’ consent to use the CQS in our research for publication in scholarly journals. Importantly, the granted permission involved creating a Polish version of the CQS. This permission obliged us to include the following copyright information on all electronic and paper copies of the survey: *Cultural Intelligence Center 2005*. *Used by permission of Cultural Intelligence Center*. *Note*: *Use of this scale granted to academic researchers for research purposes only*. *For information on using the scale for purposes other than academic research (e*.*g*., *consultants and non-academic organizations)*, *please send an email to* info@culturalq.com. This copyright information was directly translated into Polish and is used in all electronic and paper copies of the CQS. The final version of the Cultural Intelligence Scale is provided in the S1 Appendix.

#### The Cross-Cultural Competence Inventory

The Cross-Cultural Competence Inventory is a comprehensive self-report tool for measuring cross-cultural competencies (CCCI) ([29, 30] for Polish adaptation see [46]). The CCCI consists of 63 statements rated by participants on a 6-point scale (ranging from 1 = *strongly disagree* to 6 = *strongly agree*). The questions are part of 7 scale dimensions:

1. Cultural Adaptability (among others, understanding the point of view of people from a different culture and different methods of solving problems). 18 items, e.g. *A job is often successful because you understand the people you are working with well*.
2. Self-Presentation (whether a person can look straight into the eyes of another person and lie to her or cheat her). 4 items, e.g. *I'm not always the person I appear to be*.
3. Tolerance of Uncertainty (whether a person likes to change plans at the last minute). 11 items, e.g. *I don't like situations that are uncertain*.
4. Determination (concentration skills, avoiding uncertainty, being decisive). 7 items, e.g. *I would never describe myself as indecisive*.
5. Engagement (asking inter alia if a person, when feeling stressed, can calm down or think about other things). 11 items, e.g. *When feeling stressed*, *I’m able to calm myself by thinking of other things*.
6. Mission Focus (specifying whether a person can find several solutions while coping with a problem). 7 items, e.g. *I think that having clear rules and order at work is essential for success*).
7. Lie and Social Desirability Scale. 5 items, e.g. *I feel that there is no such thing as an honest mistake*.

The scale obtained satisfactory psychometric properties in previous studies: internal consistency (Cronbach’s α. .83 to .86 for the total score), test-retest reliability (.79), theoretical, criterion and convergent validity (for a further review of the Polish adaptation of the CCCI, see [47]).

#### The Emphatic Sensitiveness Scale

The Emphatic Sensitiveness Scale (ESS) [47] is a self-report tool for measuring empathy. The ESS consists of 28 items rated by participants on a 5-point scale (ranging from 1 = *strongly disagree* to 5 = *strongly agree*) and is based on the model of empathy proposed by Davis [48, 49]. The ESS consists of three components:

1. Empathic Concern (11 items; e.g. *I often have tender*, *concerned feelings for people less fortunate than me*).
2. Personal Distress (8 items; e.g. *I sometimes feel helpless when I am in the middle of a very emotional situation*).
3. Perspective Taking (9 items; e.g. *I try to look at everybody's side of a disagreement before I make a decision*).

While the first two relate to the emotional aspect, the third relates to the cognitive aspect of empathy. The reliability coefficients (internal consistency) for the ESS equalled .74–.78 and the theoretical validity was confirmed.

#### The Short version of the Need for Cognitive Closure Scale

The Short version of the Need for Cognitive Closure Scale (SNCCS) [36] is a self-report tool for measuring an individual’s preference for coming to closure in making decisions and judgements. The SNCCS consists of 15 items rated on a 6-point scale (1 = *strongly disagree*, 6 = *strongly agree*) and is based on the Need for Closure Scale by Webster and Kruglanski [50] (Polish version by Kossowska [51]). It consists of 5 domains:

1. Order (preference for order and structure). 3 items, e.g. *I think that having clear rules and order at work is essential for success*.
2. Predictability (preference for predictability of future contexts). 3 items, e.g. *I like to have friends who are unpredictable*.
3. Ambiguity (discomfort associated with the absence of closure). 3 items, e.g. *I don't like situations that are uncertain*.
4. Closed Mindedness (avoidance of alternative opinions and inconclusive evidence). 3 items, e.g. *Even after I've made up my mind about something*, *I am always eager to consider a different opinion*.
5. Decisiveness (desire to reach closure by making judgments or decisions). 3 items, e.g. *I would describe myself as indecisive*.

The reliability coefficients (internal consistency) for the SNCCS equalled .52–.86 and the theoretical validity was confirmed.

#### The International Personality Item Pool–Big Five Markers– 20

The International Personality Item Pool–Big Five Markers– 20 (IPIP-BFM-20) [52] is a self-report tool measuring the Big Five personality traits:

1. Extraversion (the level of sociability and social confidence). e.g. *(I) Am the life of the party*.
2. Agreeableness (attitude towards other people). e.g. *(I) Am interested in people*.
3. Conscientiousness (the level of diligence in organization and accomplishing goals). e.g. *[I] Am always prepared*.
4. Emotional Stability (the level of emotional reactivity and stability). e.g. *(I) Get stressed out easily*).
5. Intellect (creativity and level of openness to experience). e.g. *(I) Have a rich vocabulary*.

It consists of 20 items (5 items per each personality trait) rated on a 5-point scale (1 = v*ery inaccurate*; 5 = *very accurate*). The IPIP-BFM-20 has sufficient and satisfactory reliability coefficients ranging from .61–.82 and the theoretical validity was confirmed.

#### The Social Desirability Scale

The Social Desirability Scale [53] is a self-report tool for measuring an individual’s need to be accepted and readiness to behave in a manner that is perceived favourably by others. The scale consists of 29 items of the “true-false” type (e.g. *I am never late for school (work)*). The reliability coefficients (internal consistency and stability) of the questionnaire equalled 0.79–0.90. High coefficients of correlation (up to 0.82) with Marlowe-Crowne’s scale [54] were also obtained [53]. By using the Social Desirability Scale we wanted to control for the possibility that participants tried to deliberately express their open attitudes towards culturally diverse people to please the experimenter. The social desirability is a need to be accepted and being ready to behave in a manner that is perceived favourably by others. The issue of the need for social approval bears on the majority of interviews especially if they regard issues important for the respondent, e.g. an attitude towards people coming from other cultures and religions.

#### The Emotional Intelligence Scale

The Emotional Intelligence Scale [55] is a self-report questionnaire for measuring the level of emotional intelligence. This scale consists of 25 statements rated on a 5-point scale (1 = v*ery inaccurate*; 5 = *very accurate)* measuring the concept of emotional intelligence introduced by Salovey and Mayer [56]. It consists of three main domains:

1. empathy and perception of emotions. 9 items relating to the ability to recognize, identify and empathize with emotional states expressed by others, e.g. *I can almost always tell how my interlocutor feels*.
2. insight with emotional knowledge. 9 items relating to insight into one’s own emotions, e.g. *I am usually very clear about my feelings*.
3. mood managing. 7 items relating to the ability to manage negative emotions and states, e.g. *I have my own ways of overcoming sadness and anger*.

The Emotional Intelligence Scale has sufficient and satisfactory reliability coefficients ranging from .63–.81 and the theoretical validity was confirmed.

#### The Rosenberg Self-Esteem Scale

The Polish Rosenberg Self-Esteem Scale (SES) [42] consists of 10 items (e.g. *I feel that I have a number of good qualities*) rated on a 4-point scale (1 = *strongly disagree*; 4 = *strongly agree*) and measures global self-esteem defined as attitude towards the self. The SES has sufficient and satisfactory reliability coefficients ranging from .81–.83 and the theoretical validity was confirmed.

#### The International Personality Item Pool–Big Five Markers– 50

The International Personality Item Pool–Big Five Markers– 50 (IPIP-BFM-50) [44] is the Polish adaptation of Goldberg’s [57] IPIP-BFM-50 self-report questionnaire for measuring the five personality traits: (1) Extraversion, (2) Agreeableness, (3) Conscientiousness, (4) Emotional Stability, and (5) Intellect. It consists of 50 items (10 items per scale) rated on a 5-point scale (1 = v*ery inaccurate*; 5 = *very accurate*). The IPIP-BFM-50 has sufficient and satisfactory reliability coefficients ranging from .77–.88 and the theoretical validity is confirmed. Please note that since the aforementioned IPIP-BFM-20 is a shortened version of the IPIP-BFM-50, it has slightly lower reliability coefficients compared to the full-length prototype. Therefore, in order to increase the precision of personality assessment, we additionally decided to use the IPIP-BFM-50 during the second experimental session.

#### The Positive/Negative Attitude Towards Culturally Divergent People Questionnaire

The questionnaire consisted of 6 questions relating to the two main research areas: (1) the participant’s experience in interacting with and attitude towards people from diverse cultural backgrounds, and (2) attitude towards refugees. Regarding the former, participants were asked whether they: (a) have lived abroad for at least 1 month (Yes/No); (b) have close relationships with culturally diverse people (Yes/No); (c) would be willing (Yes/No) to marry a person from an ethnic minority (e.g. Roma, Afro-American), a different nation (e.g. German, Russian), or a religious minority community (e.g. Jehovah’s Witness, Muslim, Jewish). In addition, they decided (Yes/No) whether European, Muslims, Romas and Afro-Americans should be granted the same free health care benefits as Polish citizens. As to the second area, participants were instructed to think about refugees coming to Poland and to answer (Yes/No) whether they should be accepted by the Polish government and whether the most promising students from war-torn countries (e.g. Syria, Iraq) should be granted free medical university education. Please note that Study 1 was conducted after the European migrant crisis that occurred in 2015 when the European Commission decided to relocate Syria and Iraq refugees from south European countries to other the EU members. This matter started a long-lasting political discussion in Poland and divided the public as to the validity of the EC decision.

### Data collection

The study was conducted between December 2017 and March 2018. The Research Ethics Committee at the Institute of Psychology at Jagiellonian University approved this study. Written consent for participation was obtained prior to data collection. No incentive was offered for participation in the study. The privacy and confidentiality of participants was strictly protected as follows: (1) all the information provided by each participant was coded by a number that does not directly identify any individual; (2) any identifying information was coded and removed from all non-numerical data so it is impossible for anyone but the experimenter to identify any individual; (3) any coded identifying information was kept separately from raw questionnaires and responses; (4) if an individual chose to stop participating in the study, any data already collected was removed from the study records.

Participants from Jagiellonian University Medical College and The Medical University of Lodz were invited (either during participants’ classes or via email) by one of the two experimenters (either AM or PP, respectively) to participate in the study. Importantly, the experimenters did not recruit students from the classes they themselves taught to avoid putting any pressure on students. Participants were informed that the goal of the study was the psychometric evaluation of the Polish adaptation of tool measuring cultural competencies and intelligence. They were also told that the study was for research purposes only. In addition, participants were informed that the study consisted of two experimental sessions, each lasting up to 1 hour. It was highly stressed that the study was entirely voluntary and they would not receive incentives for their participation or individual information about their results. They were also informed that they would be able to withdraw from the study at any point without any consequences. Finally, participants were informed of how their privacy and confidentiality would be protected.

Volunteers willing to participate in the study were tested in groups by either AM (Jagiellonian University Medical College) or PP (Medical University of Lodz) during scheduled sessions that took place at the University. During each experimental session, participants were told the same information as during the recruiting phase. Importantly, they were explicitly informed that they were free to withdraw from the study at any point. The experimenter assured them that their responses would be confidential and they could refrain from reporting particularly sensitive information by marking “X” as an answer.

Since we wanted to study the relationship between cultural intelligence and the wide variety of different variables (e.g. personality, self-esteem, emotional intelligence), it was unfeasible (due to time constraints and participants’ capacity) to ask participants to fill in all the questionnaires during one session. For this reason, participants were provided with different sets of tools during the first and second sessions (but the CQS was filled in during each session). The first and second sessions lasted up to 1 hour. Below we more precisely describe the sets of tools used.

**First session.** During the first session, participants completed the following questionnaires: CQS, CCCI, The Positive/Negative Attitude Towards Culturally Divergent People Questionnaire, Emphatic Sensitiveness Scale, The Need for Closure Scale, International Personality Item Pool–Big Five Markers 20, The Social Desirability Scale.

**Second session.** During the second session they completed CQS, The Emotional Intelligence Scale, The Rosenberg Self-Esteem Scale, IPIP-BFM-50.

Finally, a new group of professional cross-cultural trainers completed only the CQS and CCCI tools during one study session.

### Statistical analysis

The software STATISTICA (version 12.00; Site License) and IBM SPSS AMOS (version 25.00) were used for statistical analysis. The licences were obtained by and granted to Jagiellonian University for research purposes and were used in accordance with the terms of use by the first author only (KB). In the descriptive statistics we used means and standard deviations. To analyse the reliability of the CQS we utilized (a) Cronbach’s alpha [58] to assess the internal consistency, (b) correlation coefficients to determine the test-retest stability of the CQS [59], and (c) confirmatory factor analysis (CFA; [60]) to confirm the postulated four-factor structure of CQS [2]. To assess the validity of the CSQ we used (a) an independent sample t-test to examine the theoretical validity [61], and (b) correlation coefficients to determine the convergent validity and criterion validity [59, 62]. For all statistical tests reported below the rejection level was set at 0.05 (unless otherwise specified, see 4.2.3). For all t-tests the effect size was measured by Cohen’s *d* with small, medium, and large effects defined as 0.2, 0.5, and 0.8, respectively [63].

## Results

### Descriptive results, reliability: Internal consistency, test-retest reliability and factorial structure

The overall means for the CQS are provided in Table 1. As can be seen, the internal consistency of the adapted CQS inventory (Cronbach’s α) was .94 and .95 in the first and second session, respectively. Importantly, the internal consistency parameter ranged between .87–.94 and .89–.95 across the subscales in the first and second session, respectively. The one-month test-retest reliability for the total score in CQS was *r*(349) = .77, *p* < .001 and it ranged between .62 and .80 across subscales.

**Table 1:** Means, standard deviations, Cronbach's α for Cultural Intelligence Scale (CQS).

	Non-Professionals: Group of non-cross-cultural trainers						Correlations Test-Retest	Professionals: Group of cross-cultural trainers		
	Session 1: Test			Session 2: Retest			CQS			
	*M*	*SD*	*Cronbach's α*	*M*	*SD*	*Cronbach's α*	*r*	*M*	*SD*	Statistics ^1^^,^ ^2^
CQS: Total score	81.81	19.90	.94	80.39	19.77	.95	*r*(349) = .77, *p < .001	97.81	17.37	*t(383)* = 4.64, *p* < .001, *q = .010, *d* = .88)
Metacognitive CQ	17.94	4.94	.88	16.90	4.67	.90	*r*(349) = .62, *p < .001	21.78	3.64	*t(383)* = 4.54, *p* < .001, *q = .020, *d* = 1.13)
Cognitive CQ	20.72	6.48	.87	20.95	6.45	.89	*r*(349) = .66, *p < .001	24.83	6.46	*t(383)* = 3.63, *p* < .001, *q = .040, *d* = .67)
Motivational CQ	21.64	6.61	.90	21.26	6.10	.91	*r*(349) = .80, *p < .001	25.33	5.05	*t(383)* = 3.26, *p* < .001, *q = .050, *d* = .72)
Behavioural CQ	21.51	6.68	.92	21.28	6.33	.94	*r*(349) = .67, *p < .001	25.86	5.32	*t(383)* = 3.76, *p* < .001, *q = .030, *d* = .72)

Notes: The average distance between test and re-test was 28.06 ±4.40 days, range = 22 to 47 days. Significant results are marked with an asterisk (e.g. *p, *q).

^1^ We compared the average results between cross-cultural trainers and non-professional participants’ results obtained during the first session.

^2^ Tests are statistically significant at the corrected *q* = .050 level.

Next, we performed confirmatory factor analysis to further examine the factorial structure of the CQS. In particular, we verified the postulated a four-factor structure of the CQS [2]. Importantly, we used the following multiple fit indices (as suggested by, for example [64, 65]) to thoroughly evaluate the model’s fit: chi-square statistic (*χ2*), normed chi-square (*χ2/df*), goodness-of-fit index (*GFI*), the adjusted goodness-of-fit index (*AGFI*), normed fit index (*NFI*), root mean square error of approximation (*RMSEA*), comparative fit index (*CFI*), non-normed fit index (*NNFI*), and standardized root mean square residual (*SRMR*). The results indicated that while the four-factor CQS model postulated by the authors did not perfectly fit the data, it was satisfactory and acceptable: *χ2*(164) = 482.56, *p* < .001, *χ2/df* = 2.94, *GFI* = .88, *AGFI* = .84, *NFI* = .90, *RMSEA* = .08, 90% *CI* (.07, .08), *CFI* = .93, *NNFI* = .92, *SRMR* = .04. For example, models with the following values indicate an acceptable fit: *CFI* values between .80 and .90 (or higher); *RMSEA* and *SRMR* values close to (or lower than) .06 and .08, respectively, and up to .10; *χ2/df* values should be less than 3; GFI, AGFI and NFI higher than .90, .95, and .90, respectively (e.g. [65, 66, 67, 68, 69]). Importantly, all the items were significantly related to the general latent trait (*ps* < .001; standardized regression weights ranging from .66 to .90).

### Validity

#### Criterion validity

In order to further examine the CQS’s criterion validity we correlated the total score of the CQS with another tool constructed to measure a similar concept; namely, the Cross-Cultural Competence Scale (CCCI) [29, 30]. Please note that the Author of the original CCCI [30, 31] recommends excluding from further analysis participants that perform higher than 15 on the “Lie and Social Desirability” scale. Therefore, while correlating the CQS with the CCCI we excluded from this analysis 46 participants and 5 participants in the non-professional and professional trainers groups, respectively. The final sample for this analysis consisted of 317 participants (284 females, 33 males) aged 18–53 (M = 21.46, SD = 4.66) in the non-professional group and 31 participants (21 females, 10 males) aged 28–65 (M = 44.51, SD = 8.51) in the professional trainers group.

As presented in Table 2, the correlation between CCCI and CQS was *r*(303) = .66, *p* < .001 and *r*(31) = .76, *p* < .001 in the non-professional group and professional trainers, respectively.

**Table 2:** Correlations of the CQS with Cross-Cultural Competence Inventory and other measures (e.g. personality, empathy).

		Non-Professionals: Group of non-cross-cultural trainers							Professionals: Group of cross-cultural trainers			
		Session 1: Test			Session 2: Retest			Correlations with CQS (total score)				Correlations CQS
		*M*	*SD*	*Cronbach's α*	*M*	*SD*	*Cronbach's α*	*r*	*M*	*SD*	*Cronbach's α*	*r*
Cross-Cultural Competence Inventory^1^		218.26	22.04	.82	N/A			*r*(303) = .66, *p < .001	236.84	21.20	.85	*r*(31) = .76, *p < .001
Emphatic Sensitiveness Scale	Empathic Concern	40.36	5.32	.73				*r*(349) = .12, *p = .028	N/A			
	Personal Distress	24.57	4.44	.65				*r*(349) = -.14, *p = .008				
	Perspective Taking	33.23	4.18	.69				*r*(349) = .32, *p < .001				
The Need for Closure Scale	Order	12.56	2.95	.77				*r*(349) = .02, *p* = .703				
	Predictability	163.29	1.89	.73				*r*(349) = -.02, *p* = .674				
	Ambiguity	13.17	2.45	.66				*r*(349) = -.06, *p* = .236				
	Closed Mindedness	7.74	2.19	.68				*r*(349) = -.41, *p < .001				
	Decisiveness	10.12	3.23	.78				*r*(349) = .11, *p = .037				
International Personality Item Pool–Big Five Markers 20	Extraversion	15.34	3.71	.83				*r*(349) = .20, *p < .001				
	Agreeableness	18.23	2.35	.67				*r*(349) = .19, *p < .001				
	Conscientiousness	15.46	3.34	.74				*r*(349) = .02, *p* = .669				
	Emotional Stability	13.04	3.08	.72				*r*(349) = .09, *p* = .104				
	Intellect	16.99	2.52	.66				*r*(349) = .37, *p < .001				
The Social Desirability Scale		15.57	4.71	.66				*r*(349) = .16, *p < .001				
Emotional Intelligence Scale	Perception of emotions and empathy	N/A			34.56	4.62	.77	*r*(349) = .30, *p < .001				
	Insight with emotional knowledge				36.11	4.15	.70	*r*(349) = .15, *p = .005				
	Mood managing				27.61	3.89	.74	*r*(349) = .22, *p < .001				
The Rosenberg Self-Esteem Scale					25.31	6.60	.86	*r*(349) = .10, *p* = .058				
IPIP-50	Extraversion				3.32	0.74	.90	*r*(349) = .18, *p < .001				
	Agreeableness				3.92	0.53	.82	*r*(349) = .25, *p < .001				
	Conscientiousness				3.52	0.58	.81	*r*(349) = .14, *p = .008				
	Emotional Stability				2.82	0.70	.88	*r*(349) = .07, *p* = .165				
	Intellect				3.58	0.51	.76	*r*(349) = .36, *p < .001				

Notes:

^1^We analysed results for the CCCI after excluding participants performing highly on the Lie and Social Desirability scale. Significant results are marked with an asterisk (e.g. *p).

#### Convergent validity

As demonstrated in Table 2, CQS positively correlated with: (1) emphatic sensitiveness–perspective taking and empathic concern; (2) need for cognitive closure–decisiveness; (3) social desirability; and (4) emotional intelligence–empathy and perception of emotions, insight with emotional knowledge and mood managing. At the same time, it was negatively correlated with: (1) emphatic sensitiveness–personal distress; (2) need for cognitive closure–closed mindedness. Finally, the cultural intelligence scale positively correlated with personality traits such as extraversion, agreeableness, consciousness (IPIP-BFM-50 but not IPIP-BFM-20), and intellect. We did not find any significant correlations between CQS and self-esteem, emotional stability, the need for order, predictability and ambiguity tolerance.

#### Theoretical validity

To analyse the CQS’s validity we verified whether participants who demonstrated positive relationships with and/or attitudes towards foreign-born populations, minorities and migrants performed higher on the CQS scale, as it may be theoretically expected. For example, we would expect that an individual who has a close and positive relationship with the Roma minority would perform higher on the cultural intelligence scale compared to someone who has no such experience. To fulfil this goal, we conducted a series of independent t-tests for differences in the total score of CQS between participants with positive and negative attitudes that were operationalized as agreeing (positive attitude) or disagreeing (negative attitude) with statements such as, for example, “Refugees from Syria or Iraq should be provided with free university education”. In total, we performed 15 t-tests. To control for multiple comparisons we chose the False Discovery Rate correction [70]. With *α* = .05, the critical value *q* was .043.

As can be seen in Table 3, participants who declared a positive attitude towards culturally diverse groups of people obtained significantly higher scores on the CQS. The only non-significant difference was between people who were for or against providing EU citizens and Afro-Americans with health care benefits within the Polish healthcare system. More precisely, independently of being for or against culturally-diverse people, participants were equal in terms of the average total score in the CQS (a medium to large effect sizes).

**Table 3:** Means and standard deviations for CQS total scores across participants with either positive or negative attitude towards foreign residents (e.g. refuges, immigrants, foreign-born people).

		Attitude toward foreign residents and minorities				
		Positive		Negative		
		*M*	*SD*	*M*	*SD*	Statistics
Having a close/friendly relationship with culturally divergent people		90.13	18.90	76.61	18.74	*t(347)* = 6.53, *p* < .001, *q = .003, *d* = .72)
Living abroad for at least a month in the past		88.51	20.48	80.18	19.44	*t(347)* = 3.14, *p* = .002, *q = 033., *d* = .42)
Would you be willing to marry:	German	85.29	19.70	71.80	19.15	*t(274)* = 4.57, *p* < .001, *q = .013, *d* = .69)
	Muslim	90.44	21.53	79.22	19.50	*t(283)* = 2.82, *p* = .005, *q = .037, *d* = .55)
	African-American	87.26	18.96	71.97	19.70	*t(260)* = 5.77, *p* < .001, *q = .007, *d* = .79)
	Russian	87.04	19.73	74.73	18.39	*t(262)* = 4.66, *p* < .001, *q = .010, *d* = .65)
	Roma	88.80	20.66	77.85	18.96	*t(251)* = 3.69, *p* < .001, *q = .020, *d* = .55)
	Jehovah's Witness	88.57	21.54	78.25	18.77	*t(260)* = 3.46, *p* < .001, *q = .023, *d* = .51)
	Jew	85.38	20.59	75.30	18.37	*t(251)* = 3.82, *p* < .001, *q = .017, *d* = .52)
Should refugees from Syria and Iraq be accepted by the Polish government?		88.88	20.91	78.98	19.07	*t(259)* = 3.21, *p* < .001, *q = .027, *d* = .49)
Granting free university education to refugees from Syria and Iraq		84.81	19.39	78.74	20.37	*t(266)* = 2.26, *p* = .025, *q = .043, *d* = .31)
Granting free health care to:	Europeans	82.33	20.26	84.50	14.96	*t(322)* = .30, *p* = .763, *q* = .050, *d* = .12)
	Muslims	86.53	20.25	77.56	18.25	*t(256)* = 3.14, *p* < .001, *q = .030, *d* = .47)
	Romani people	85.21	20.52	77.38	18.39	*t(256)* = 2.41, *p* < .017, *q = .040, *d* = .40)
	African-Americans	84.62	20.68	78.21	18.16	*t(265)* = 1.82, *p* = .070, *q* = .047, *d* = .33)

Note. Tests are statistically significant at the corrected *q* = .043 level. Significant results are marked with an asterisk (e.g. *q). Positive attitude indicated being open to culturally divergent groups of people (e.g. agreeing to provide them with medical education free of charge).

Finally, to test the differences between professionals and non-professionals in the CQS, the overall means for the CQS total score as well as for the CQS’s subscales were entered into an independent *t-test*. With *α* = .05, the critical corrected value *q* was .050. As can be seen in Table 1, compared to non-professional participants, the professional cross-cultural trainers scored significantly higher on the CQS in general, and on all subscales in particular (a medium to large effect size).

## Discussion

The present study examined the psychometric properties of the Polish version of the Cultural Intelligence Scale (CQS), developed originally by Ang et al. [2]. More precisely, we assessed both the reliability (i.e. internal consistency, test-retest reliability, factor structure) and validity (i.e. theoretical, criteria, convergent). We discuss these psychometric properties in the relevant sections below.

### The general reliability of the Cultural Intelligence Scale: Internal consistency, factorial structure, test-retest reliability

The first goal of our study was to analyse the reliability of the CQS. For this reason, participants completed the CQS twice, which additionally allowed us to verify the test-retest reliability. Our findings provide strong evidence that CQS has satisfactory internal consistency (i.e. Cronbach’s alpha ranged between .88–.95) and is quite stable over time (i.e. the test-retest correlations ranged between .62 and .80).

At the same time, although the original version of the CQS was assumed to have a 4-dimensional factor structure [2], confirmatory factor analysis did not provide strong evidence for this assumption. More precisely, this factor structure did not fit the empirical data perfectly, but the fit was still acceptable. This finding accords well with results obtained by Bücker et al. [3, 71] and Ward et al. [72], thus suggesting that a two-factor structure represents the empirical data better than the postulated four-factor CQS model. More precisely, in their study [3] they proposed a new two-dimensional CQS model in which metacognitive and cognitive items are combined into a new single dimension called ‘*internalized cultural knowledge intelligence’*, and motivational and behavioural items are combined into second single dimension called ‘*effective cultural flexibility intelligence*’. As suggested by Bücker et al. [3], while the former relates to one's awareness of cultural knowledge, expressed by both awareness of cultural knowledge and cultural knowledge *per se*, the latter reflects the self-conscious adjustment that comprises both the self-efficacy and the ability to (non-)verbally adjust to culturally divergent situations and contexts.

In summary, while there is a need to further investigate the factorial structure of the CQS, since all items were significantly and strongly related to the general latent trait, we argue that the CQS can be successfully used to measure the general concept of cultural intelligence. Put differently, we highly recommend the usage of the total score of the CQS.

### The general validity of the Cultural Intelligence Scale: Theoretical, criterion and convergent validity

The second overriding goal of the present study was to examine the validity of the CQS. To fulfil this aim, we examined theoretical validity, criterion validity and convergent validity.

Regarding criterion validity, it was expected that CQS would correlate highly with another tool constructed to measure the theoretically similar concept of cross-cultural competencies. Our results were in line with this expectation; namely, we observed a statistically significant high positive correlation with the Cross-cultural Competence Inventory (CCCI) [29, 30]. Therefore, we successfully proved the criterion validity of the CQS.

Next, we examined the theoretical validity of the CQS in two ways: Firstly, we compared the group of non-professionals with the group of cross-cultural-trainers in terms of their mean total score in the CQS. As expected, we observed higher cultural intelligence scores for professionals compared to non-professionals. Secondly, participants who demonstrated a positive attitude to culturally diverse national and religious groups of people scored significantly higher on the CQS. These findings together provide strong empirical support for the theoretical validity of the CQS.

Finally, we also assessed the convergent validity of the CQS by examining the correlations between CQS and the following variables: (1) personality–extraversion, agreeableness, conscientiousness, intellect (positive correlation); (2) empathic sensitiveness–perspective taking and empathic concern (positive correlation), personal distress (negative correlation); (3) the need for cognitive closure–decisiveness (positive correlation), closed mindedness (negative correlation); (4) emotional intelligence–perception of emotion and empathy, insight with emotional knowledge and mood managing (positive correlation); (5) social desirability (positive correlations). As expected and argued in the present study section, variables such as personality, emotional intelligence and empathy (i.e. empathic concern and perspective taking) correlated positively with cultural intelligence, while closed mindedness and personal distress were negatively correlated. In general, these findings suggest that the development of cultural intelligence and efficient and appropriate interaction with culturally diverse individuals may be supported by (a) being open to new and novel situations and experiences; (b) the ability to recognize and feel the emotions expressed by others; (c) the ability to communicate with people who have different ways of functioning and emotional expression; (d) the ability to take different perspectives and points of view; (e) the ability to empathize with others; (f) being resistant to anxiety or discomfort when observing others’ negative experiences; (g) having positive attitudes towards other people; (h) having a high level of sociability.

Importantly, these results are in line with findings reported in the literature [38, 43, 73, 74] which show that the aforementioned variables are important predictors of cultural intelligence and therefore should be taken into consideration when preparing programs for developing and fostering cultural intelligence. These results also give us an interesting insight into the factors that significantly influence cultural intelligence.

### The Cultural Intelligence Scale as an assessment tool for healthcare professionals

While, it can be argued that the development of cultural intelligence is an important developmental task for every person who has contact with foreigners, in the present paper we placed the focus of our attention mainly on healthcare professionals. In the present form of the education of medical professionals, students of medical majors are more focused on the development of social skills. However, a current challenge for medicine and nursing is to develop cross-cultural intelligence and competencies in order to meet the demands and needs of the global community. If there were to be a significant increase in the number of culturally diverse patients, difficulties could arise that are related to different ways of communication (verbal, non-verbal), approach to time, problem solving, decision making or other interpersonal relations. Therefore, cultural intelligence should become an important attribute of all employees, including healthcare professionals who take up jobs in culturally diverse environments that allow cultural differences to be overcome. Cultural Intelligence is a “signpost” to understanding the norms adopted in a different culture–it helps when applying culturally specific rules of behaviour to situations that require them. It is not about forcing oneself into an artificial imitation of behaviours and attitudes; it is about adjusting one’s behaviour towards culturally diverse patients/co-workers so that they do not feel discomfort in our presence. It seems that CQS can be widely used as a tool for studying cultural intelligence. For example, CQS allows predictions to be made related to vulnerability to cultural shock and labour productivity [75], cultural adaptation [76, 77], travel stress [78], psychological adaptation, and social and cultural adaptation [79]. In addition, CQS can be successfully used in empirical research among medical professionals (e.g. doctors, nurses) and students of medical majors. Finally, the CQS seems to be a useful tool for the education of future medical professionals. For instance, it allows measurement and assessment of the ability and preparedness to care for culturally divergent patients.

### Possible limitations and future directions

When considering the findings of the present study, some limitations should be taken into account. For instance, since the CFA acceptably but not perfectly confirmed the 4-dimensional structure of the CQS (but it still bears a resemblance to this postulated structure), future studies should further address the factor structure of the CQS. At the same time, as argued above, usage of the total CQS score is still highly recommended. Importantly, the next step that needs to be taken is to examine the factor structure of the Polish version of the CQS using exploratory factorial analysis (i.e. EFA). While this exceeds the scope of the present paper, we argue that this is already one of the most important and urgent issues that need to be addressed in future studies.

Second, the possible relationship between cultural intelligence and other variables (such as personality or professional competences) still needs to be thoroughly examined. More precisely, since cultural intelligence may be considered as an important factor for maintaining efficient social interactions with culturally and ethnically diverse people, it may also influence the labour productivity of nurses and other medical professionals; however, this assumption still needs empirical verification. There is also a need for studies that more thoroughly examine discriminant (i.e. divergent) validity by establishing the relationships between CQS scores and other tests measuring variables that should not directly affect cultural intelligence, such as general cognitive capability (e.g. Raven’s Advanced Progressive Matrices, as in Ward et al. [72]).

Third, it is worth highlighting that all measures discussed in the present study were based on self-report, which may put some constraints on our data. As pointed out elsewhere [46], it may be argued that responses given by participants were influenced by social desirability, namely the need to be accepted and the readiness to behave in a manner that is perceived favourably by others. As a result, participants might have declared a higher cultural intelligence than they really had. The observed weak but statistically significant positive correlation between the Social Desirability Scale and the CQS lends some support to this possible limitation. However, in order to minimize this type of bias, participants were reassured about the confidentiality of their responses and were encouraged to answer honestly. Importantly, this issue pertains not only to this single study, but also to a more general limitation regarding studies using questionnaires. It also pertains to broader questions about the extent to which self-reports correspond to real-life behaviour. To address this limitation, future studies could examine how differences in CQS are reflected by real-life decisions and behaviours (e.g. the way an individual responds to culturally diverse patients or clients).

Next, while the sample of the population of healthcare professionals, medical students and student nurses were recruited from two large Universities in Poland, we are aware that there is still a need for further studies including larger sample sizes of such participants from different Universities in general, and from different regions in particular. It might also be useful to control participants’ clinical experience with and without culturally divergent clients to additionally evaluate the role of previous clinical experience in the development of cultural intelligence. What is more, cultural intelligence might also be further evaluated among different groups of professionals (e.g. police officers, teachers). Finally, one may argue that analysing the differences in the CQS between the professional cross-cultural trainers group and the non-professional participant group may not be very informative, mainly due to the small sample size of the cross-cultural trainers. Ideally, future studies should further examine the theoretical validity by using a comparable sample size of both professional cross-cultural trainers and non-professionals.

Taking all these together, as is evident from the foregoing discussion, our results open up a set of intriguing questions for future research.

### Final conclusions

Due to the fact that until recently Poland was a relatively homogeneous country, its inhabitants did not have to work in a culturally diverse milieu. Developing skills such as cultural intelligence was a neglected area in education, including medical education and research. Despite the possible limitations of the presented CQS research tool, it should be considered important and reliable and it can be used to assess cultural intelligence among medical professionals and students of medical majors in Poland. Importantly, it has been shown in the described studies that CQS has satisfactory psychometric properties. It has high reliability and the factor structure seems to approach the postulated one. Theoretical and criterion accuracy has been well proven; although convergence is less straightforward, it correlates well with research tools and variables such as cultural competence, cognitive closure, empathy/emphatic sensitiveness, emotional intelligence, self-esteem, personality, social desirability. Therefore, it may be argued that these factors contribute to the development of cultural intelligence. Finally, this tool can be used, e.g. before and after intercultural training of medical students, doctors, nursing educators and clinical managers, and it helps to identify areas of professional competence in which development of cultural intelligence is necessary to improve the quality of care.

## Supporting information

S1 Appendix The Polish version of the Cultural Intelligence Scale. (PDF)

S2 Appendix Data base of Study. (XLSX)
