# Supplementary material for: The Polish version of the Cultural Intelligence Scale: Assessment of its reliability and validity among healthcare professionals and medical faculty students
Source: PLoS One. 2019 Nov 25;14(11):e0225240. doi: 10.1371/journal.pone.0225240 (PMC6876986; doi:10.1371/journal.pone.0225240)
Supplement: S1 Appendix — (PDF) [file pone.0225240.s001.pdf]

Skala Inteligencji Kulturowej

Pseudonim:……………………………………..……………… Płeć:…………………. Wiek:……………….

Wykształcenie:………………………………………..……………… Zawód:…………………………………………….
Email/dane kontaktowe (opcjonalne): …………………………………………… Narodowość ……………………………………

Instrukcja: Zapoznaj się uważnie z poniższymi stwierdzeniami, a następnie określ, w jakim stopniu zgadasz się z każdym z nich. Do każdego stwierdzenia należy ustosunkować się korzystając z następującej skali:

Zdecydowanie nie zgadzam się						Zdecydowanie zgadzam się
1	2	3	4	5	6	7

Przeczytaj każde stwierdzenie i wybierz taką odpowiedź, która najlepiej opisuje to, jaka/jaki NAPRAWDĘ JESTEŚ. Nie ma tutaj odpowiedzi dobrych i złych, dlatego odpowiadaj szczerze i zgodnie z własnym przekonaniem.

Zaznacz swoje odpowiedzi poprzez zakreślenie kółkiem cyfry w odpowiedniej kratce.

		Zdecydowanie nie zgadzam się						Zdecydowanie zgadzam się
1.	Jestem świadoma/y wiedzy kulturowej, której używam podczas komunikowania się z osobami z różnych kultur.	1	2	3	4	5	6	7
2.	Aktualizuję swoją wiedzę kulturową, kiedy komunikuję się z osobami z nieznanej mi kultury.	1	2	3	4	5	6	7
3.	Jestem świadoma/y swojej wiedzy kulturowej, którą stosuję w interakcjach międzykulturowych.	1	2	3	4	5	6	7
4.	Sprawdzam dokładność swojej wiedzy kulturowej, kiedy komunikuję się z osobami z różnych kultur.	1	2	3	4	5	6	7
5.	Znam prawne i ekonomiczne systemy obowiązujące w innych kulturach.	1	2	3	4	5	6	7
6.	Znam zasady (np. słownictwo, gramatykę) obowiązujące w innych językach.	1	2	3	4	5	6	7
7.	Znam wartości kulturowe i przekonania religijne istniejące w innych kulturach.	1	2	3	4	5	6	7
8.	Znam zwyczaje dotyczące zawierania małżeństw, które istnieją w innych kulturach.	1	2	3	4	5	6	7
9.	Znam sztukę i rzemiosło innych kultur.	1	2	3	4	5	6	7

Wersja oryginalna kwestionariusza: Cultural Intelligence Center 2005. Wykorzystane za zgodą Cultural Intelligence Center. Uwaga. Zgoda na wykorzystanie tej skali została przyznana Zespołowi tylko do celów badawczych. W celu uzyskania informacji na temat wykorzystania skali do celów innych niż badania akademickie (np. doradztwo zawodowe, organizacje pozaakademickie), należy wysłać maila na adres: info@culturalq.com. **Wersja polska kwestionariusza:** wykorzystywana i/lub reprodukowana za zgodą autorów: Barzykowski, K., Majda, A., Szkup, M., & Przyłęcki, P. (2019). The Polish version of the Cultural Intelligence Scale: Assessment of its Reliability and Validity Among Healthcare Professionals and Medical Faculty Students. PLOS ONE.

		Zdecydowanie nie zgadzam się						Zdecydowanie zgadzam się
10.	Znam zasady wyrażania zachowań niewerbalnych, które obowiązują w innych kulturach.	1	2	3	4	5	6	7
11.	Lubię nawiązywać kontakty z osobami z różnych kultur.	1	2	3	4	5	6	7
12.	Jestem przekonana/y, że potrafię nawiązać towarzyskie kontakty z lokalnymi mieszkańcami, którzy pochodzą z nieznanej mi kultury.	1	2	3	4	5	6	7
13.	Jestem przekonana/y, że potrafię poradzić sobie ze stresem związanym z dostosowaniem się do kultury, która jest dla mnie nowa.	1	2	3	4	5	6	7
14.	Lubię żyć w kulturach, które są dla mnie obce.	1	2	3	4	5	6	7
15.	Jestem przekonana/y, że mogę przyzwyczaić się do zasad robienia zakupów, jakie obowiązują w innej kulturze.	1	2	3	4	5	6	7
16.	Zmieniam swoje zachowanie werbalne (np. akcent, ton głosu), gdy wymaga tego interakcja międzykulturowa.	1	2	3	4	5	6	7
17.	Inaczej stosuję przerwy w mówieniu i ciszę po to, by dostosować się do różnych sytuacji międzykulturowych.	1	2	3	4	5	6	7
18.	Zmieniam tempo mówienia, gdy wymaga tego międzykulturowa sytuacja.	1	2	3	4	5	6	7
19.	Zmieniam swoje zachowanie niewerbalne, gdy wymaga tego sytuacja międzykulturowa.	1	2	3	4	5	6	7
20.	Zmieniam swoją mimikę twarzy, gdy wymaga tego interakcja międzykulturowa.	1	2	3	4	5	6	7

Wersja oryginalna kwestionariusza: Cultural Intelligence Center 2005. Wykorzystane za zgodą Cultural Intelligence Center. Uwaga. Zgoda na wykorzystanie tej skali została przyznana Zespołowi tylko do celów badawczych. W celu uzyskania informacji na temat wykorzystania skali do celów innych niż badania akademickie (np. doradztwo zawodowe, organizacje pozaakademickie), należy wysłać maila na adres: info@culturalq.com. **Wersja polska kwestionariusza:** wykorzystywana i/lub reprodukowana za zgodą autorów: Barzykowski, K., Majda, A., Szkup, M., & Przyłęcki, P. (2019). The Polish version of the Cultural Intelligence Scale: Assessment of its Reliability and Validity Among Healthcare Professionals and Medical Faculty Students. PLOS ONE.
